# Proteomic Analysis of Silk Fibroin Reveals Diverse Biological Function of Different Degumming Processing From Different Origin

**DOI:** 10.3389/fbioe.2021.777320

**Published:** 2022-02-07

**Authors:** Yaling Wang, Yunyun Liang, Jiacen Huang, Yisheng Gao, Zhixin Xu, Xuejun Ni, Yumin Yang, Xiaoming Yang, Yahong Zhao

**Affiliations:** ^1^ Key Laboratory of Neuroregeneration of Jiangsu and Ministry of Education, Co-innovation Center of Neuroregeneration, NMPA Key Laboratory for Research and Evaluation of Tissue Engineering Technology Products, Nantong University, Nantong, China; ^2^ School of Pharmacy, Nantong University, Nantong, China; ^3^ School of Public Health, Nantong University, Nantong, China; ^4^ Affiliated Hospital of Nantong University, Nantong University, Nantong, China

**Keywords:** silk, silk fibroin, silk sericin, degum, proteomic

## Abstract

Silk, as a kind of natural fibrin, has been prepared into various biomaterials due to its excellent biocompatibility and mechanicalness. However, there are some controversies on the biocompatibility of silk fibroin (SF), especially when it coexists with sericin. In this study, two kinds of silk from Jiangsu and Zhejiang were degummed with two concentrations of Na_2_CO_3_ solution, respectively, to obtain four kinds of silk fibroin. The effects of different degumming treatments on silk fibroin properties were analyzed by means of color reaction, apparent viscosity measurement, and transmission electron microscope and isobaric tags for relative and absolute quantification analyses, and the effects of different silk fibroin membranes on the growth of Schwann cells were evaluated. The results showed that the natural silk from Zhejiang treated with 0.05% Na_2_CO_3_ solution had a fuller structure, higher apparent viscosity, and better protein composition. While SF obtained by degumming with 0.5% Na_2_CO_3_ solution was more beneficial to cell adhesion and proliferation due to the thorough removal of sericin. This study may provide important theoretical and experimental bases for the selection of biomaterials for fabricating artificial nerve grafts.

## Introduction

Peripheral nerve repair has been one of the difficult problems in the field of neuroscience, especially the repair of long-distance serious injury. With the development of tissue engineering, artificial nerve grafts are becoming more and more popular. It is expected to replace autologous nerve transplantation (the “gold standard” at present) to repair long-distance injury because it can make up for the shortage of autologous nerve transplantation, such as limited source, tissue size and structure mismatch, long-term denervation, and secondary injury of the donor site (Martins et al., 2013; Ring, 2013; Castillo-Galvan et al., 2014).

In the past few decades, a large number of natural or synthetic materials, such as chitosan (Wang et al., 2005; Demina et al., 2017; Yu et al., 2017), silk fibroin (Baecker et al., 2017; Luo and Shao, 2017), collagen (Sayanagi et al., 2020), and poly (lactic-co-glycolic acid) (Wang et al., 2014), have been used to prepare artificial nerve grafts.

Among numerous biomaterials, silk, one of the earliest animal proteins, was first used in the textile industry and as surgical suture. Silk is a kind of natural high-molecular-protein polymer, which is synthesized in the special glands of the epithelial cells of silkworms, secreted into the cavity, and finally spun into fibers. It mainly contains silk fibroin (SF) and sericin. SF is in the middle of the silk, surrounded by sericin (Minoura et al., 1995). The content of SF accounts for most of the silk, about 70–80%, and sericin accounts for about 20–30%.

*In vivo* and *in vitro* studies have shown that SF without sericin will not cause obvious inflammation (Wang et al., 2008; Yang et al., 2007a). Therefore, SF-based biomaterials have been widely used in bone, tendon, and nerve repair and other tissue engineering fields due to their favorable biocompatibility, robust mechanical properties, physicochemical properties, and biological activities (Gotoh et al., 2004; Dal Pra et al., 2005; Bhardwaj and Kundu, 2012; Guptaa et al., 2013). In terms of sericin, it was not well investigated during the past decades and was simply discarded as waste in traditional silk reeling industry (Lamboni et al., 2015). Fortunately, in recent years, people have gradually realized that sericin is a kind of polymer material with specific biological properties (Yun et al., 2016). It facilitates cell adhesion and can inhibit cell apoptosis, can promote cell differentiation (Song et al., 2016), and has been used in the fabrication of a variety of biomaterials, such as thin films (Zhang et al., 2015), hydrogels (Siritientong et al., 2011; Tao et al., 2019), and scaffolds (Dash et al., 2009).

Early studies have reported the biocompatibility of silk. It is generally believed that sericin has certain immunogenicity (Dewair et al., 1985; Wen et al., 1990), which is not conducive to its application as a biomaterial *in vivo*. In recent years, with the progress of science and technology and further research on sericin, researchers believe that sericin itself will not cause a strong immune rejection (Jiao et al., 2017). When sericin was co-cultured with mouse macrophages (RAW264.7), the mRNA expression levels of IL-1 β and TNF-α were lower than those in negative control group, which proved that sericin did not cause an immune response (Panilaitis et al., 2003). Some people think that only the co-existence of sericin and silk fibroin can produce immunogenicity, but the two alone will not (Yang et al., 2007b; Teuschl et al., 2014). Therefore, different silk and degumming degrees will have different effects on the properties and biocompatibility of silk fibroin.

In this paper, silk from Jiangsu and Zhejiang were degummed with 0.05% Na_2_CO_3_ and 0.5% Na_2_CO_3_, respectively, to obtain four kinds of SF, and the antherea silk was taken as the control group. The composition and structure differences of each sample and its influence on cell growth were analyzed. The study is anticipated to provide important theoretical and experimental bases for the selection of biomaterials for artificial nerve graft preparation.

## Materials and Methods

### Materials

*Bombyx mori* silk was purchased from Jiangsu and Zhejiang (China). Dulbecco’s modified Eagle’s medium and fetal bovine serum were obtained from Gibco (United States). Forskolin, heregulin, cytosine arabinoside, and 488-labeled goat anti-mouse IgG were obtained from Sigma-Aldrich (United States). Rabbit anti-S100 beta monoclonal antibody was obtained from Abcam. CCK-8 kit was purchased from Ribobio (Guangzhou, China).

### Degumming of Silkworm Raw Silk

Fresh silkworm raw silk from different sources (Jiangsu and Zhejiang) was weighed and put into a sodium carbonate solution with different concentrations (0.05 and 0.5 wt%) in proportion. The silk was boiled three times in Na_2_CO_3_ solution (half an hour each time). The degummed silk was thoroughly rinsed with Millipore water and air-dried on a super clean platform to obtain refined silk, that is, silk fibroin sample for standby. There are 4 samples in total: the raw silk from Jiangsu degummed with 0.05 and 0.5% Na_2_CO_3_ are sample 1 and sample 2, respectively, while the raw silk from Zhejiang degummed with 0.05 and 0.5% Na_2_CO_3_ are sample 3 and sample 4, respectively. The tussah silk degummed with 0.5% Na_2_CO_3_ is labeled as sample 5.

### Dissolution of Silk Fibroin

The refined silk of silkworm was dissolved in a ternary solvent system of CaCl_2_/H_2_O/C_2_H_5_OH (mole ratio, 1:8:2) at 75 ± 2°C and then dialyzed against Millipore water in a cellulose tube (molecular cutoff = 12,000–14,000) at room temperature for 3 days.

### Degumming Degree Test

Picric acid–carmine staining was used to evaluate the degumming degree of silk degummed with two kinds of Na_2_CO_3_ solution (Zhang et al., 2014). First, in preparing the staining solution, carmine was dissolved in 25% ammonia, followed by adding saturated picric acid aqueous solution and adjusting the pH to 8.0–9.0. Then, the refined bombyx silk samples were immersed in the staining solution in test tubes, and the tubes were heated in boiling water bath for 5 min. Lastly, the samples were thoroughly rinsed with ddH_2_O and air-dried. Raw silk was taken as the control.

### Determination of Apparent Viscosity

The refined samples were dissolved in a tertiary solvent system of CaCl_2_/H_2_O/C_2_H_5_OH (mole ratio, 1:8:2) and then kept in water bath at 20°C for 2 h. Apparent viscosity was then measured with NDJ-7 rotary viscometer.

### Transmission Electron Microscopy Observation

Samples under different degumming treatments were fixed in pre-cooled 2.5% glutaraldehyde, post-fixed in 1% osmium acid, dehydrated with gradient ethanol, embedded in EPON 812 epoxy resin, and cut into slices of 70-nm thickness. The ultra-thin sections were stained with lead citrate and uranium acetate, followed by observation under a transmission electron microscope (JEOL Ltd., Tokyo, Japan). In Photoshop 7.0, the fiber diameter was measured by measuring tools. The diameter of at least 60 fibers was measured from 10 photos taken in different fields randomly. The average value and standard deviation of the fiber diameter were calculated.

### Isolation of Schwann Cells

In this study, all experimental procedures involving animals were conducted as per the institutional animal care guidelines and approved ethically by the administration committee of experimental animals, Jiangsu Province, China.

Schwann cells (SCs) were harvested as described previously (Chen et al., 2017). Briefly, the sciatic nerves and dorsal root ganglia were isolated from neonatal Sprague–Dawley rats (1 to 2 days) to get primary rat Schwann cells. The obtained tissues were triturated and enzymatically digested with 0.25% trypsin at 37°C for 30 min. Then, the mixture was centrifuged and re-suspended in Dulbecco’s modified Eagle’s medium (DMEM) supplemented with 10% fetal bovine serum (FBS), followed by plating on poly-l-lysine pre-coated dishes. After incubation for 24 h, cytosine arabinoside was added to allow cell incubation for another 24 h to remove fibroblasts. Next, the cells were cultured in DMEM supplemented with 10% FBS, 2 mM forskolin, and 2 ng/ml heregulin to stimulate cell proliferation. When cells covered 90% of the dish surface, they were further purified with anti-Thy1 antibody (1:1,000, AbD Serotec, Raleigh, NC, United States) and complement (Jackson Immuno, West Grove, PA). Lastly, the purified SCs were cultured in DMEM with FBS and growth factor until the cells were sufficient to seed on the SF membrane.

### Schwann Cells Culture

The prepared SF membranes were sterilized with 75% alcohol for 30 min and rinsed extensively with sterilized phosphate-buffered saline (PBS). Then, all samples were put in 24-well culture plates, and cell suspension was added in. The seeding cell density was 1 × 10^5^ cells/well. At different times of culture, the morphological changes of Schwann cells on these different SF membranes were observed under an inverted light microscope.

### CCK-8 Assay

CCK-8 kit was used to evaluate the viability and proliferation of SCs on different samples after culturing for 1 and 3 days, respectively. At different times, fresh DMEM medium with CCK-8 reagent (*V*_medium_ / *V*_CCK-8_ = 10:1) was added to replace the cell medium to allow cell incubation at 37°C for 4 h. Then, 150 μl of suspension was transferred to a 96-well plate. The absorbance was measured at 450 nm by an ElX-800 micro-ELISA reader (Bio-Tek Inc., United States).

### Immunostaining of Schwann Cells

The morphology of Schwann cells on different samples was examined by the immunostaining method. In brief, after 2 days of culture, cells were rinsed with PBS for three times thoroughly; then, they were fixed in 4% paraformaldehyde at 4°C for 4 h and stained with S100 (1:400) at 4°C for 24 h, followed by further reaction with IgG-488 (1:400) at 37°C for 2 h. Subsequently, the cells were incubated with Hoechst (final concentration: 5 μg/ml) at room temperature for 15 min. Finally, cell samples were observed under an immuno-fluorescence microscope (Leica, Germany).

### Calcein AM/PI Test

The viability of Schwann cells was evaluated with Calcein-AM/PI Double Staining Kit (Invitrogen, L3224). Schwann cells were seeded on four different silk fibroin membranes and control plates at a density of 1 × 10^5^ cells/well. After the SCs were cultured for 1 and 3 days, DMEM with 10% FBS was discarded, and the samples were rinsed with PBS. Then, calcein-AM and propidium iodide (PI) were added, and the cells were incubated at 37°C for 30 min. Images were captured by an immuno-fluorescence microscope.

### Proteomic Quantitative Analysis of Different Silk Fibroin Samples

To find out the different proteins between samples and select the appropriate silk materials, we used isobaric tags for relative and absolute quantification (iTRAQ) to analyze proteins in different samples quantitatively (Zou et al., 2019). The phenol extraction method was used for protein extraction, which can effectively remove the small molecular interference in the sample. At the same time, filter-aided proteome preparation enzymolysis strategy was used for protein digestion. Then, the peptides of each sample were labeled with iTRAQ, and the labeled samples were graded by high-pH reversed-phase classification strategy. Finally, data collection of samples obtained by classification was carried out by the ultra-high-resolution mass spectrometer Q-Exactive.

### Statistical Analysis

The statistical significance was analyzed by GraphPad Prism 7.0 (GraphPad Software, Inc.). One-way ANOVA followed by Tukey’s *post-hoc* test was used to compare individuals among different groups of the same time. Two-way analysis of variance (two-way ANOVA), followed by Sidak’s multiple-comparisons test, was employed when comparing −ES and +ES groups of all groups. Data were presented as mean ± SD. *p* < 0.05 was considered statistically significant.

## Results

### Effect of Degumming Method on Degumming Degree

**FIGURE 1:**
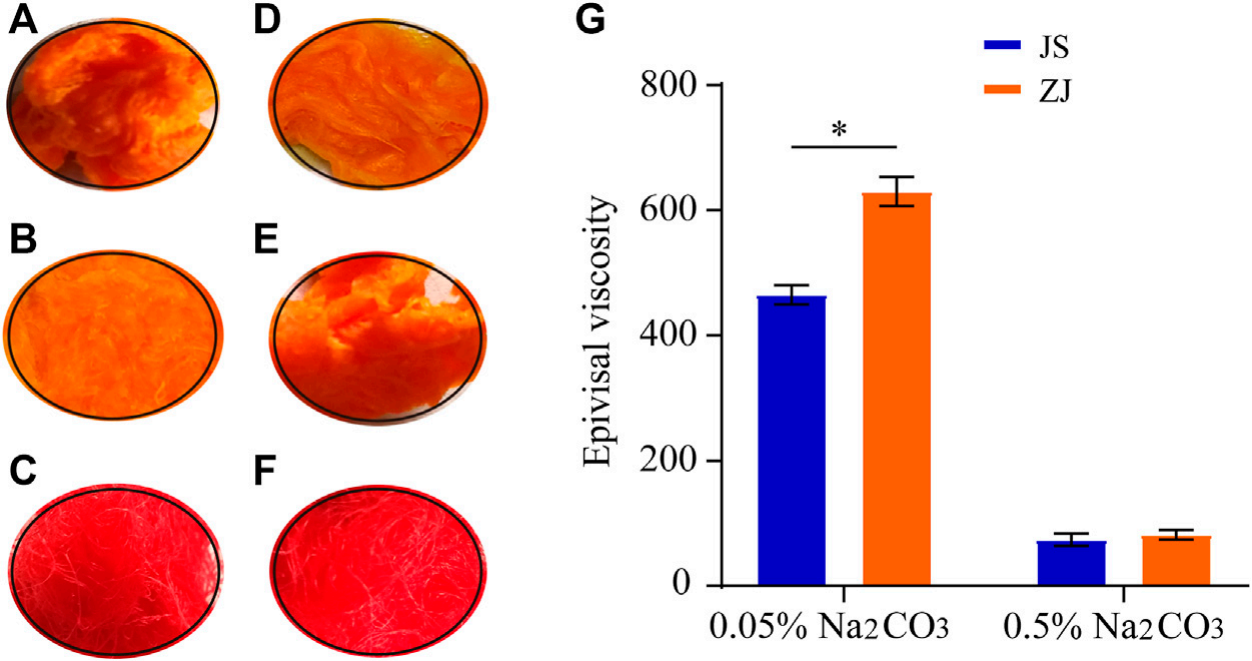
Optical images of dyed silkworm silk from different sources, degummed by Na_2_CO_3_ with different concentration **(A–F)** and epivisal viscosity **(G)**, *p < 0.05. Silk from Jiangsu degummed by 0.05% Na_2_CO_3_ **(A)** and 0.5% Na_2_CO_3_ **(B)**; no degumming treatment **(C)**. Silk from Zhejiang degummed by 0.05% Na_2_CO_3_ **(D)** and 0.5% Na_2_CO_3_ **(E)**; no degumming treatment **(F)**.

Two kinds of Na_2_CO_3_ solution (0.05 and 0.5 wt%) were chosen to degum silk from different sources. All silk from different sources in the two solutions were boiled for 0.5 h, and the test was repeated three times. The degumming degree of two kinds of Na_2_CO_3_ solutions on the raw silk from different sources was determined by the picric acid–carmine staining method. SF and sericin have different absorbance capacity on picric acid and carmine. Silk fibroin turns yellow in alkaline solution due to its selective adsorption of picric acid. However, sericin has a strong capacity to absorb both picric acid and carmine; red covers yellow, so it appears red. Therefore, after dyeing and washing, the yellow surface of raw silk indicates that sericin has been completely removed; otherwise, it indicates that sericin has not been completely removed. It can be seen from Figure 1 that, after degumming with the Na_2_CO_3_ solution, samples 1–4 are yellow (Figures 1A,B,D,E), indicating that sericin has been removed, while the control group without degumming can be clearly observed to be red (Figures 1C,F). With the increase of Na_2_CO_3_ solution concentration, the samples appear pure yellow (Figures 1B,E), showing that the degumming degree is higher.

### Effect of Silkworm Raw Materials on Apparent Viscosity

The SF obtained by different degumming methods was dissolved in ternary solution in the same proportion, and its apparent viscosity was measured (Figure 1G). The viscosity of Zhejiang silk decreased from 630 to 78 CP, while that of Jiangsu silk changed from 450 to 75 CP as the concentration of Na_2_CO_3_ changed from 0.05 to 0.5%, indicating that the high concentration of Na_2_CO_3_ can catalyze the degradation or hydrolysis of SF. Under the catalysis of the high concentration of Na_2_CO_3_, the molecular chain of SF becomes shorter, the number of entangled nodes in the solution decreases, and the friction resistance between molecules decreases, which leads to the decrease of viscosity. Therefore, according to the viscosity change, the concentration of Na_2_CO_3_ should be reduced as much as possible to reduce the damage to the viscosity of SF. At the same time, we also found that the viscosity of the SF solution of Zhejiang silkworm was higher than that of Jiangsu silkworm under two kinds of Na_2_CO_3_ concentration.

### The Effect of Degumming Method on the Structure of Silk Fibroin

**FIGURE 2:**
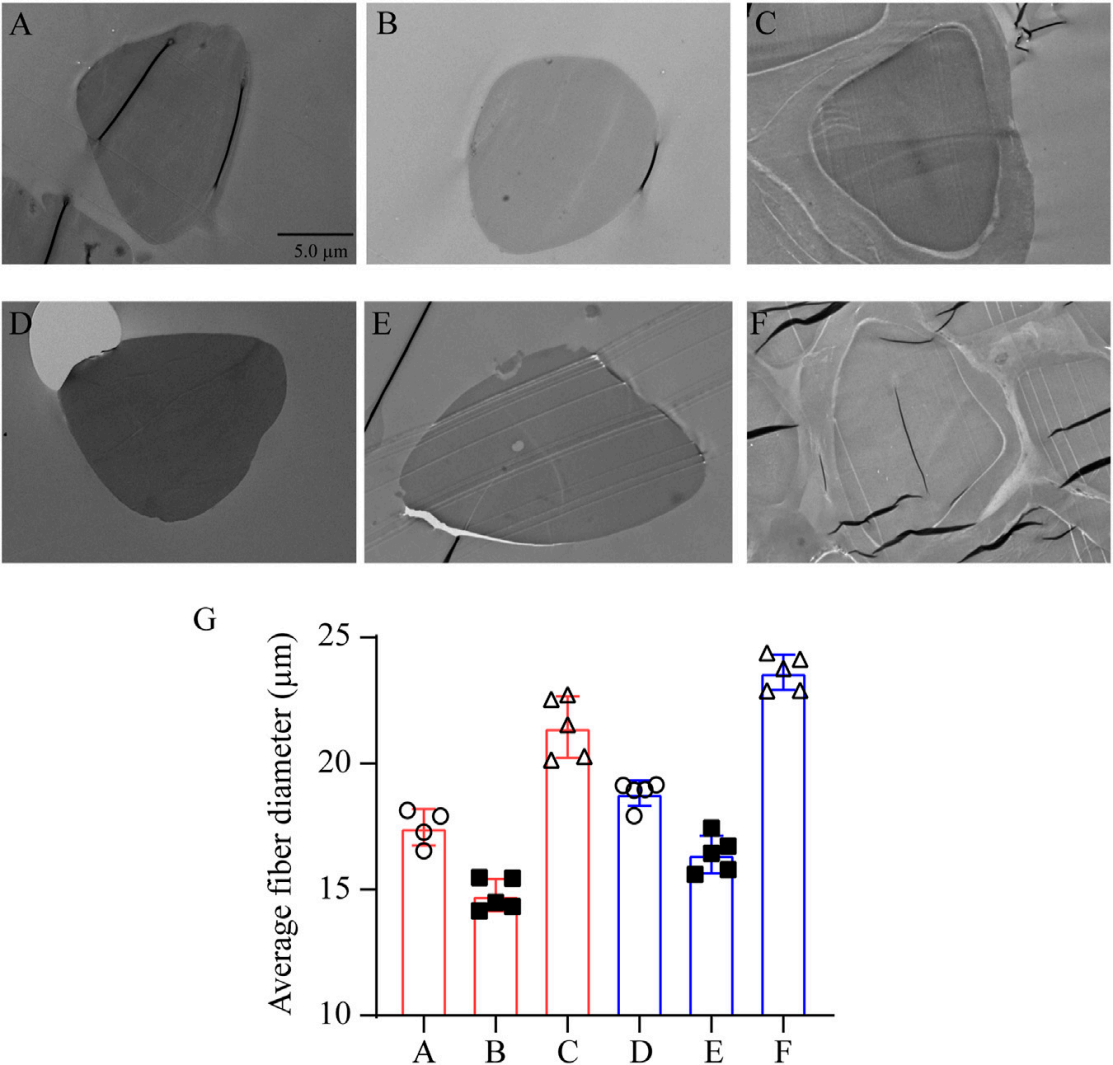
Transmission electron microscopy of silkworm silk from different sources, degummed by Na_2_CO_3_ with different concentration. Silk from Jiangsu degummed by 0.05% Na_2_CO_3_ **(A)** and 0.5% Na_2_CO_3_ **(B)**; no degumming treatment **(C)**. Silk from Zhejiang degummed by 0.05% Na_2_CO_3_ **(D)** and 0.5% Na_2_CO_3_ **(E)**; no degumming treatment **(F)**. The parts circled in yellow in samples **(C)** and **(F)** are marked as the sericin layer. **(G)** Statistical graph of the fiber diameter of **(A–F)**. **(A–F)**: bar = 5 μm.

Silk from different sources was degummed with different concentrations of Na_2_CO_3_ solution. After degumming with 0.05% Na_2_CO_3_, the cross-section of the SF sample demonstrates a homogeneous oval with complete structure, smooth edge, and no attachment on the surface, indicating that 0.05% Na_2_CO_3_ can not only remove sericin on the surface but also maintain the structure of SF. While treated with 0.5% Na_2_CO_3_, the section of degummed SF presents oval of different sizes and a long fusiform shape. It appears wrinkled with incomplete edge, and the diameter gets smaller, demonstrating that the high concentration of Na_2_CO_3_ makes sericin completely removed, but it also destroys the structure of SF to some extent. Comparing the effect of source on the structure of SF, despite the concentration of the Na_2_CO_3_ solution, the cross-section diameter of SF obtained from Zhejiang silkworms was larger than that from Jiangsu silkworms, indicating that the structure of Zhejiang silkworms was relatively full (Figure 2).

### Morphology of Schwann Cells

Schwann cells play an important role in the formation of myelin sheath during peripheral nerve regeneration. Therefore, in this study, the adhesion, survival, and growth of Schwann cells on different silk fibroin membranes were studied to choose a suitable SF to prepare peripheral nerve grafts which can better promote peripheral nerve regeneration.

**FIGURE 3:**
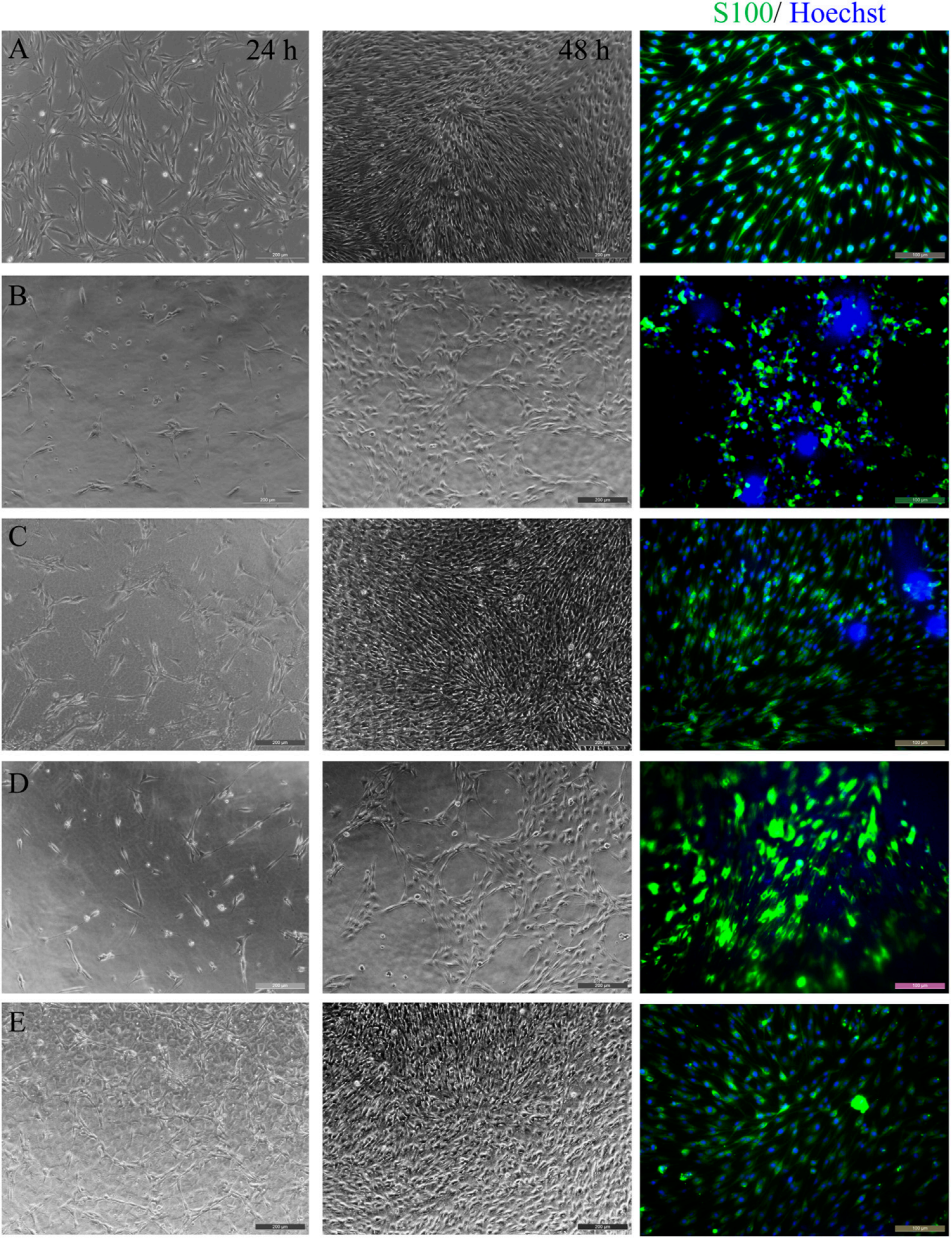
Morphology observation of Schwann cells on different samples for 24 and 48 h. Brightfield images (two columns to the left) and immunofluorescence staining (column to the right, 48 h). **(A)** control, **(B)** JS-0.05, **(C)** JS-0.5, **(D)** ZJ-0.05, **(E)** ZJ-0.5. Scale bar, 50 μm.

Figure 3 shows the morphology observation of SCs on different samples for 24 and 48 h, respectively. After 1 day of culture, there were certain amounts of cell on all samples, but the number of cells on JS-0.5 and ZJ-0.5 was more than that on JS-0.05 and ZJ-0.05, and some cells on JS-0.05 and ZJ-0.05 shrank into a circle, indicating that cells could grow on all samples, but samples JS-0.5 and ZJ-0.5 were more conducive to cell adhesion. It may be that a small amount of residual sericin in JS-0.05 and ZJ-0.05 affected the interaction between cells and samples, resulting in inferior number and the morphology of cells on JS-0.05 and ZJ-0.05. Two days later, the cell density on sample JS-0.5 and ZJ-0.5 increased, indicating good cell growth and proliferation, and the number of cells on samples JS-0.05 and ZJ-0.05 increased, too, but not as much as that of JS-0.5 and ZJ-0.5, indicating that JS-0.05 and ZJ-0.05 can also support cell growth and promote cell proliferation, but the cell growth was slower than that of JS-0.5 and ZJ-0.5. The results of immunofluorescence staining tells us that, on samples JS-0.5 and ZJ-0.5, SC cells exhibited better adhesion and spreading and displayed a spindle-like shape with filopodia at both ends, while on sample JS-0.05 and ZJ-0.05 the cell bodies were spindle or round, which is consistent with the brightfield images. Moreover, the morphology and quantity of the cells on ZJ-SF were better than those on JS-SF.

### Survival and Proliferation of Schwann Cells on Different Silk Fibroin Membranes

**FIGURE 4:**
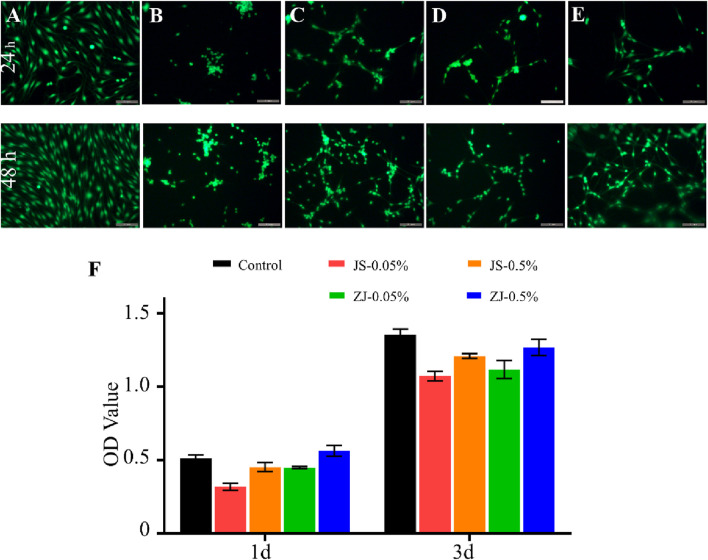
Viability of Schwann cells on different samples for 24 h and 48 h. Images of calcein-AM/propidium iodide (PI) double-staining: **(A)** control, **(B)** JS-0.05, **(C)** JS-0.5, **(D)** ZJ-0.05, and **(E)** ZJ-0.5. Scale bar, 50 mm. Green fluorescence indicates live cells stained with calcein-AM and red fluorescence indicates dead cells stained with PI. Scale bar, 200 μm. **(F)** CCK-8 test of Schwann cells.

The viability and proliferation of Schwann cells on all samples were evaluated by calcein-AM/PI double staining and CCK-8 test, respectively. The results are shown in Figure 4. It was found that Schwann cells can grow on all samples, and almost all cells are green (Figure 4A), indicating that all samples can support cell survival and growth. Both staining test and CCK-8 test (Figure 4B) showed that the number of cells increased over time, which further proved that all the samples exhibited no cytotoxicity and supported cell proliferation. However, the number of cells on the sample made of silk fibroin degummed with 0.05% Na_2_CO_3_ was relatively larger, as the thoroughly degummed silk fibroin membrane was more conducive to cell adhesion. In addition, under the same degumming conditions, compared with JS-SF, the number of cells on the ZJ-SF samples is relatively more in 1 or 3 days, and the morphology of the cells was better.

### Proteome-Wide Identification and Classification of Different Samples

**FIGURE 5:**
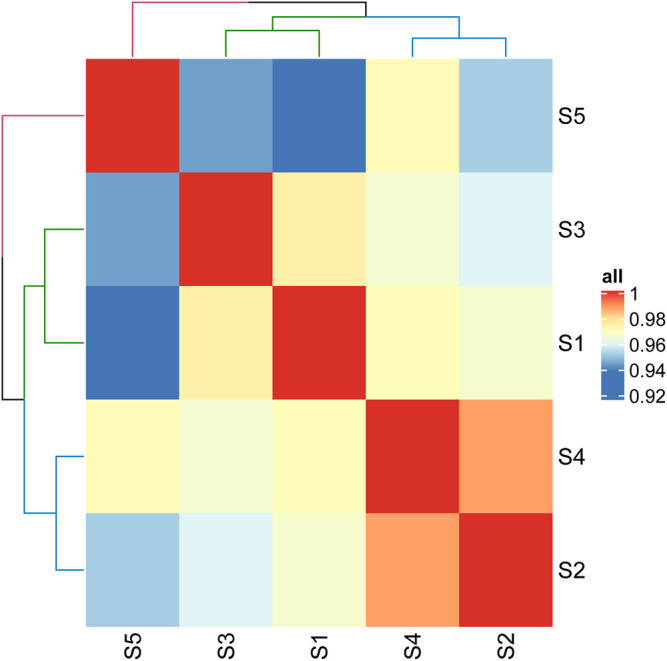
Correlation heat map of different samples. S1: JS-0.05, S2: JS-0.5, S3: ZJ-0.05, S4: ZJ-0.5, S5: *Antheraea pernyi* silk.

In order to find out the reasons for the differences of cell adhesion and growth on different products, we analyzed the protein of samples and found that there were 55 kinds of proteins which were classified according to their function. Figure 5 shows the correlation analysis of each sample. It can be seen that the correlation between sample JS-0.05 and sample ZJ-0.05 is very high, as well as that between sample JS-0.5 and ZJ-0.5, indicating that the silk fibroin composition of different sources treated with the same degumming method is similar. The sample *Antheraea pernyi* silk is different from the other samples and is closest to sample ZJ-0.5, which tells us that the protein composition of Zhejiang silk treated with 0.5% is the most similar to that of *A. pernyi* silk.

Both *A. pernyi* silk fibroin and mulberry silk fibroin are fibrous proteins, and the category of amino acids is almost identical. However, there are obvious differences in quantity (Kim et al., 2012). The amino acid residues in the silk fibroin of *A. pernyi* are large, and the side chain contains rich active groups, including aspartic acid (ASP) and arginine (Arg), which can form a special RGD tripeptide sequence with glycine. Thus, *A. pernyi* silk is favorable to cell adhesion (Kopp et al., 2020), and its elongation and elasticity are higher than that of silkworm silk. Therefore, in our quantitative analysis, tussah silk was selected as the control. Through iTRAQ analysis, the silk with similar properties as tussah silk was determined so as to find the suitable silk source and degumming treatment method. According to the above-mentioned results, sample ZJ-0.5 is similar to sample *A. pernyi* silk, that is, the silk sample from Zhejiang Province being degummed in 0.5% Na_2_CO_3_. Previous cell experiments show that sample ZJ-0.5 is the best for cell adhesion, viability, or proliferation, which is well explained by the sample protein clustering here.

**FIGURE 6:**
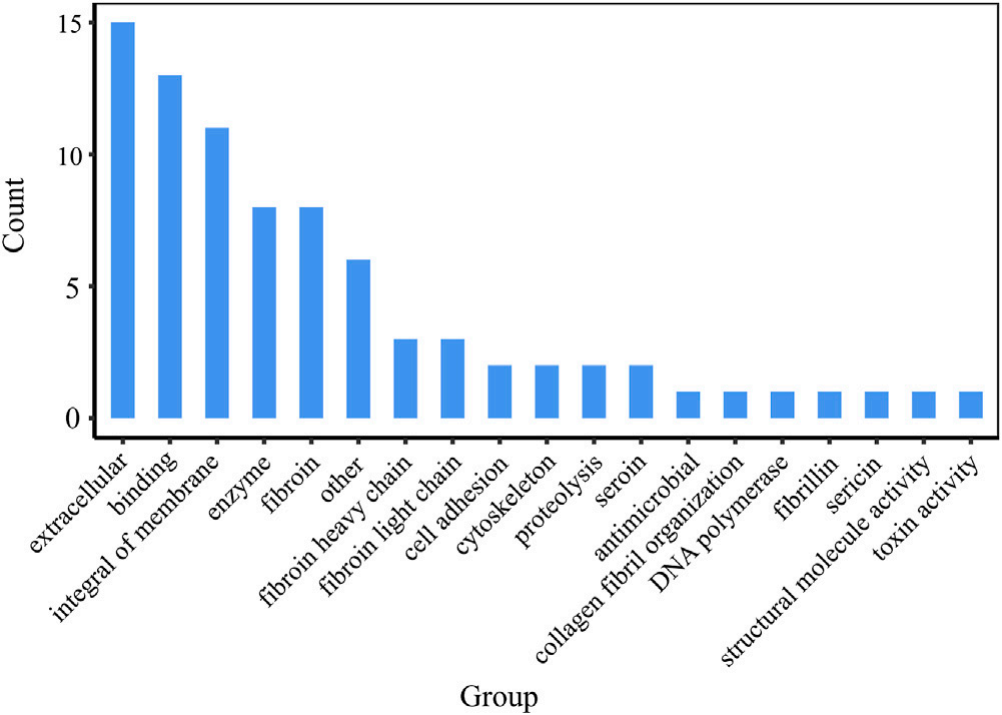
Classification of proteins in silk fibroin.

The 55 proteins identified in the samples could be classified into several categories based on their annotated molecular function: extracellular (16), binding (13), antimicrobial (1), fibroin (8), cell adhesion (2), integral of membrane (11), sericin (1), cyclosketen (2), and so on (Figure 6). It can be found that, except silk fibroin and sericin, other proteins are closely related to cell growth, which is one of the reasons that silk fibroin has good biological activity and is widely used in the biomedical field.

### Comparison of the Relative Abundance of Proteins in the Samples

**FIGURE 7:**
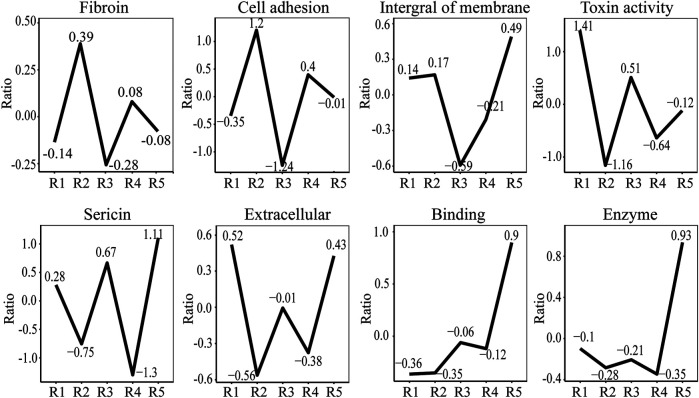
Ratio of representative proteins in different samples. R1: JS-0.05, R2: JS-0.5, R3: ZJ-0.05, R4: ZJ-0.5, R5: *Antheraea pernyi* silk.

To compare protein abundances across different samples, we used scale function to realize data standardization. Figure 7 shows that the content of silk fibroin in samples JS-0.5 and ZJ-0.5 is higher, while the sericin content is lower, which is consistent with the degumming treatment method. Samples JS-0.5 and ZJ-0.5 were treated with 0.5% Na_2_CO_3_, and sericin is removed more thoroughly. Cell adhesion and DNA polymerase are also higher in samples JS-0.5 and ZJ-0.5, and there is more toxin-activity protein in samples 1 and 3, explaining why the cells on samples JS-0.5 and ZJ-0.5 survived more and grew better. However, the contents of cytoskeleton, binding, and extracellular proteins in samples JS-0.05 and ZJ-0.05 are higher than those in samples JS-0.5 and ZJ-0.5, indicating that a high concentration of Na_2_CO_3_ can effectively remove sericin but also make silk fibroin lose some useful proteins. Therefore, the content of each protein in every sample is different. They play various roles in the process of cell adhesion and proliferation. We need to consider their effects on cell growth comprehensively, retaining as much natural matrix components as possible on the premise of thoroughly removing sericin.

## Discussion

A high concentration of Na_2_CO_3_ solution can remove sericin more thoroughly, but it also destroys the structure of SF to a certain extent, resulting in the incomplete edge of SF and the decrease in cross-sectional diameter. At the same time, SF is degraded to a great extent and more peptide bonds, hydrogen bonds, and other secondary bonds are destroyed, forming shorter protein molecular chains and making the structure of SF loose and extended. Therefore, the viscosity of SF degummed with a high concentration of sodium carbonate is smaller.

Since the residual sericin around the core SF is considered to be the source of silk-related undesirable immune reactions, it is important to develop silk purification procedures by removing sericin thoroughly and retaining SF. In this study, Na_2_CO_3_ solution with two different concentrations was chosen to remove sericin.

As a kind of glial cells, Schwann cells can not only guide the growth of regenerated axons to establish a precise innervation (Xu et al., 2013) but also secrete various neurotrophic factors, cell adhesion molecules, that are conducive to nerve regeneration (Guenard et al., 1992). Therefore, it is significant to figure out the growth of cells on silk fibroin scaffold. For this reason, we chose two different sources of silk and used different degumming methods to obtain different SF, and then SCs were co-cultured with membranes prepared with these SF.

On the SF-0.5% (SF treated with 0.5% Na_2_CO_3_) membrane, the cell survival rate was high and the cells took the standard spindle shape, showing a good growth situation. This is because SF obtained by degumming with 0.5% Na_2_CO_3_ was purer, and there was no inflammatory reaction caused by residual sericin. Therefore, SF-0.5 promotes cell adhesion and supports cell proliferation.

In order to further explore the effect of different SF samples on cell survival and growth, we used iTRAQ technology to analyze the protein composition of different samples and took *A. pernyi* silk fibroin as a reference standard. *A. pernyi* silk contains a special RGD tripeptide sequence which is beneficial to cell adhesion and gives *A. pernyi* silk better elongation and elasticity. The iTRAQ results showed that the composition of ZJ-0.5 is the closest to that of *A. pernyi* silk. Therefore, ZJ-0.5 should be the best choice to support cell adhesion, proliferation, and growth, which is consistent with the results of the cell experiment.

## Conclusion

In this paper, silk fibroin from different sources were treated with different degumming methods, their morphology, viscosity, and protein composition were analyzed, and silk fibroin membranes were prepared to co-culture with SC cells. The results indicated that the natural silk from Zhejiang Province that was treated with 0.05% Na_2_CO_3_ solution had a fuller structure, higher apparent viscosity, and better protein composition, while SF obtained by degumming with 0.5% Na_2_CO_3_ solution was more beneficial to cell adhesion and proliferation due to the thorough removal of sericin. Moreover, the different growth status of cells on different samples was explained by proteomic analysis. Overall, this study may offer an important basis for the construction of nerve conduit with suitable biomaterials.

## Data Availability

The original contributions presented in the study are included in the article/Supplementary Material. Further inquiries can be directed to the corresponding authors.
