# Correction: Proteomic analysis of silk fibroin reveals diverse biological function of different degumming processing from different origin

**DOI:** 10.3389/fbioe.2025.1612862

**Published:** 2025-06-12

**Authors:** Yaling Wang, Yunyun Liang, Jiacen Huang, Yisheng Gao, Zhixin Xu, Xuejun Ni, Yumin Yang, Xiaoming Yang, Yahong Zhao

**Affiliations:** ^1^ Key Laboratory of Neuroregeneration of Jiangsu and Ministry of Education, Co-Innovation Center of Neuroregeneration, NMPA Key Laboratory for Research and Evaluation of Tissue Engineering Technology Products, Nantong University, Nantong, China; ^2^ School of Pharmacy, Nantong University, Nantong, China; ^3^ School of Public Health, Nantong University, Nantong, China; ^4^ Affiliated Hospital of Nantong University, Nantong University, Nantong, China

**Keywords:** silk, silk fibroin, silk sericin, degum, proteomic

In the published article, there was an error in Figure 4 as published. The images were mistakenly selected from a different experimental condition than the one described in the corresponding figure legend and main text. The error occurred during the figure assembly process, as the original image files for multiple experimental conditions were stored in the same folder without distinct subcategorization. The corrected Figure 4 and its caption appear below.

**FIGURE 4:**
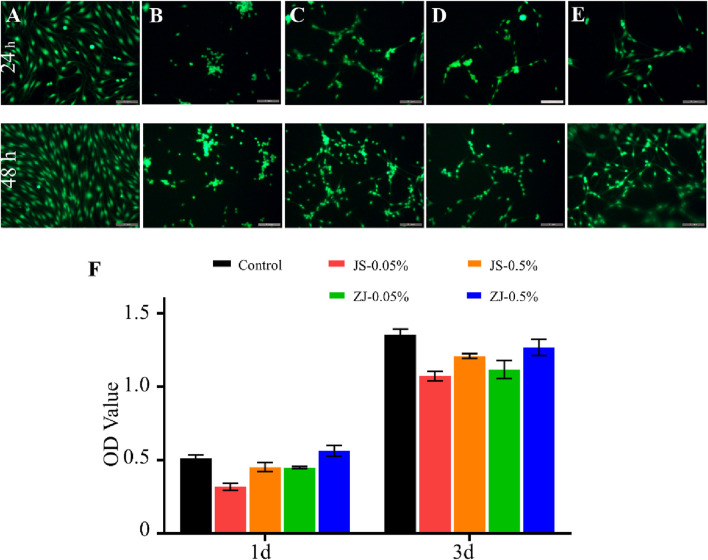
Viability of Schwann cells on different samples. Images of calcein-AM/propidium iodide (PI) double-staining: **(A)** control, **(B)** JS-0.05, **(C)** JS-0.5, **(D)** ZJ-0.05, and **(E)** ZJ-0.5. Scale bar, 50 mm. Green fluorescence indicates live cells stained with calcein-AM and red fluorescence indicates dead cells stained with PI. Scale bar, 200 μm. **(F)** CCK-8 test of Schwann cells.

The authors apologize for this error and state that this does not change the scientific conclusions of the article in any way. The original article has been updated.

